# Assessing ‘connective tissue’ in public-private partnerships: a stakeholder survey on multisectoral collaboration in global health

**DOI:** 10.1186/s12992-025-01156-x

**Published:** 2025-11-11

**Authors:** Gavin Allman, Sumithra Krishnamurthy Reddiar, Carrie Ngongo, Meritxell Mallafré-Larrosa, Cristina Parsons Perez, Helen McGuire, Roberto F. Iunes, Andrea Vassalotti, Kyle Peterson, Rachel Nugent

**Affiliations:** 1 Global Health Division, RTI International, 3040 E Cornwallis Rd, Research Triangle Park, North Carolina, NC 27709 USA; 2 Consultant, Texcoco, Mexico; 3 Department of Global Health, University of Washington, Seattle, WA USA; 4 City Cancer Challenge, Geneva, Switzerland; 5 NCD Alliance, Geneva, Switzerland; 6 Noncommunicable Diseases Program, PATH, Seattle, WA USA; 7 Health, Nutrition, and Population Global Practice, World Bank, Washington, DC USA; 8 World Heart Federation, Geneva, Switzerland; 9 Boldly Go Philanthropy, Chevy Chase, Maryland USA; 10 Health Finance Institute, Washington, DC USA

**Keywords:** Measurement, Collective action, Collaboration, Partnership, Multisectoral, Stakeholders, Noncommunicable disease, SDGs, Social capital

## Abstract

**Background:**

Public-private partnerships have the potential to advance solutions to complex dilemmas such as the prevention and control of noncommunicable diseases. Knowledge creation, trust, and social capital among partners – encapsulated in the term “connective tissue” – are key considerations for the cohesion and sustainability of multisectoral collaborative efforts in global health.

**Methods:**

A survey was conducted with 23 stakeholders of projects in four countries supported by Access Accelerated, a collective of biopharmaceutical and life sciences companies. The survey elicited perspectives on the factors that strengthen collaboration and develop knowledge creation, trust, and social capital within the multisectoral partner network.

**Results:**

Survey respondents related how connective tissue was cultivated through implementation of multiple projects with shared goals. Identified barriers to effective collaboration included resource constraints, while facilitators included shared objectives and overlapping activities. Qualitative responses provide deeper understanding of how multisectoral collaboration contributed to the sustainability of the Access Accelerated initiative.

**Conclusion:**

Measurement of connective tissue enhances understanding of project performance by addressing dynamic and previously overlooked outcomes of multisectoral collaboration. Multisectoral health initiatives can incorporate implementation science methods into measurement approaches to strengthen connective tissue among partners and stakeholders.

**Clinical trial number:**

Not applicable.

**Supplementary Information:**

The online version contains supplementary material available at 10.1186/s12992-025-01156-x.

## Background

### Introduction

Most impact evaluations in global health assess a program’s ability to reach stated objectives within a specific timeframe. Aspects of governance, collaboration, and the accrued benefits from building ties among partners and stakeholders are infrequently measured, yet these are defining traits of multisectoral health projects. Implementation science, the study of methods to promote the adoption and integration of evidence-based practices and interventions, can complement traditional measurement and evaluation of multisectoral initiatives by identifying factors that influence the strength and sustainability of collaborative partnerships. Understanding barriers and facilitators of effective collaboration among stakeholders can allow for these factors to be introduced into measurement frameworks and intentionally cultivated within multisectoral initiatives.

To bridge this gap in measuring the facilitators of effective multisectoral partnerships, we propose the concept of “connective tissue” – a term that encapsulates the knowledge creation, trust, and social capital that arise from multiple partner efforts directed toward unified goals. We propose this concept to capture the less tangible impacts of multisectoral collaboration, including those that build coherence in workstyles and that contribute to more enduring impact. This study introduces the concept of connective tissue to help explain how strong partnerships are built and, through a qualitative survey of implementer perspectives, explores the value of connective tissue to strengthen partner networks and facilitate project success. We test the concept of “connective tissue” by analyzing dynamics between strategic partners of Access Accelerated, a multisectoral partnership between biopharmaceutical companies and global stakeholders to improve the quality and accessibility of care for noncommunicable diseases in over 30 low- and middle-income countries. Survey respondents weigh in on the concept of connective tissue, identify barriers and facilitators to its development, and provide testimony of their experiences within the Access Accelerated partner network.

As international assistance for health decreases and the Sustainable Development Goals (SDGs) approach their 2030 sunset, qualitative measurement of partner networks and stakeholder engagement within multisectoral initiatives like Access Accelerated contributes to a more robust understanding of past efforts and informs future planning and prioritization. As new global targets are set and new health initiatives are founded in the coming years, the principles of connective tissue and methods from implementation science can complement existing measurement frameworks for multisectoral and whole-of-society efforts by encouraging measurement of dynamic factors that affect collaboration within partner networks.

### The multisectoral partnership

Access Accelerated is a multisectoral partnership founded by biopharmaceutical and life sciences companies in 2017 to address the burden of disease caused by noncommunicable diseases (NCDs) in low- and middle-income countries. Access Accelerated aligns its work with the SDGs, particularly the advancement of targets 3.4 (to reduce premature mortality from NCDs) and 3.8 (to achieve universal health coverage, financial risk protection, and access to safe, affordable, effective, and quality medicines and vaccines). It aims to contribute to these targets, as well as to SDG 17 (strengthen the means of implementation and revitalize the global partnership for sustainable development), by pooling funds and cultivating strong stakeholder networks to drive impact at national level [1].

Access Accelerated employs “convening power to connect stakeholders” and creates “multisectoral partnerships that can bring about real and lasting change.” [2] Funding for the initiative comes from participating pharmaceutical and life science companies, and is allocated by the Access Accelerated secretariat through a closed proposal process to five strategic partners: City Cancer Challenge (C/Can), the NCD Alliance (NCDA), PATH, the World Bank, and the World Heart Federation (WHF). Funding is provided to these five strategic partners by the Access Accelerated secretariat with the overall aim of generating evidence to catalyze additional investment and buy-in for a collective response to NCDs. Each of the strategic partners leverages its own technical strengths in capacity-building, advocacy, and health systems strengthening to implement individual projects that drive progress towards a common agenda. 1 The research firm RTI International was contracted by Access Accelerated in 2021 to measure partner projects’ individual and collective impact, as well as system-level change catalyzed by the initiative at large.

Critically, all projects are funded by Access Accelerated are conducted in collaboration with national institutions including Ministries of Health and finance to ensure alignment with existing country-level NCD efforts. For instance, evidence generation and collaborative engagement from multiple strategic partners contributed to the development of Kenya’s *National Strategic Plan for Prevention and Control of NCDs 2021/22–2025/26* [3]. The national plan for NCDs drew from an NCD investment case and pilot study of integrated care produced by the World Bank, as well as from the NCD Navigator, a digital information system that PATH and Kenya’s Ministry of Health developed with Access Accelerated funding to map NCD programs, stakeholders, and funding at the country level. Further, training and advocacy prioritization from NCDA to their Kenyan affiliate organization NCDAK resulted in lived experience representatives gaining a permanent seat on the technical working group for the development of the strategic plan.

Strategic partner support for the development of Kenya’s national plan for NCDs illustrates how targeted investment in complementary efforts can lay the foundation for collaborative engagement and contribute to outcomes that exceed the scope of any individual project. In short, that is what connective tissue tries to measure and explore from an implementation science perspective. Access Accelerated provides an example of a network of partners coordinating actions to achieve shared goals, which allowed us to study how knowledge creation, trust, and social capital have been cultivated within the partnership over time and supported the achievement of shared goals.

### Implementation science and the measurement of collective efforts

In proposing the concept of connective tissue to measure the strength of partnerships and illustrate the value of collaboration, we start from the premise that common objectives among stakeholders can best be achieved through joint efforts. However, multilateral implementation of joint efforts can introduce new challenges. In *The Logic of Collective* Action, Mancur Olson describes the free rider problem, whereby some partners may contribute more than others while yielding the same benefits. [4] Other stumbling blocks can include competition for resources, self-interested motivations, and a lack of trust between partners [5]. Olson observed that group members will work together when benefits from active participation accrue to all partners, but a coordinating body is needed to ensure that partners work harmoniously to achieve collective good [4].

Political scientist Elinor Ostrom describes conditions which enable successful and sustainable collective action: governance mechanisms, establishing processes and rules of cooperation with clear boundaries, and methods for conflict resolution [6]. From the domains or organizational management and business, Kania and Kramer pioneered the term *collective impact* to represent “the commitment of a group of important actors from different sectors to a common agenda for solving a specific social problem [7]”. They suggest five key conditions for successful collective impact to occur: 1) a common agenda, 2) shared measurement systems, 3) mutually reinforcing activities, 4) continuous communication, and 5) a backbone support organization [8, 9].

Coordination among diverse stakeholders enables collective action campaigns to target ambitious objectives, but complex and changing dynamics among partners have the potential to detract from the effectiveness and sustainability of these multisectoral initiatives [10]. Methods and perspectives from implementation science can identify and forestall such issues through evidence-based practices in partnership development, governance, and measurement. Woulfe et al. cite key factors for effective multisectoral partnership including leadership, organizational structure, member selection, resource management, common vision, and relations among stakeholders [11]. A 12-country study series by Hinton et al. identified common indicators of success, sector-specific research questions, adaptability and mixed methods measurement as best practices for multisectoral collaboration [12]. Glandon et al. point to ongoing implementation science research gaps for effective multisectoral collaboration, such as the role of power dynamics, classification of governance arrangements, indicators of collaboration, and exploration of the perspectives and experiences of partners [13].

Insights on sustainable multisectoral partnership strengthening have taken root among development practitioners and become reflected in program measurement. *Communication for Social Change: an integrated model for measuring the process and its outcomes* from Johns Hopkins University Center for Communication Programs focuses on qualitative indicators, such as “what was done to get stakeholders and beneficiaries involved in the program” and “what are the mechanisms being used for all community members to communicate their interests” [14]. Similarly, academics and environmentalists in Australia developed the *Community Engagement for Collective Action: a handbook for practitioners*, which emphasizes an introspective measurement approach centered on stakeholder engagement [15].

This analysis of connective tissue builds on previous research on collective efforts from the fields of sociology, political science, organizational management, implementation science, and development practice. It reaffirms assertions from prior scholarship that governance structures, stakeholder engagement mechanisms, and shared definitions of project success influence the quality of collaboration within multisectoral partnerships. What distinguishes the concept of connective tissue from the body of existing research is the recognition that multisectoral collaboration is influenced not only by immutable and foundational qualities of a partnership, but also by evolving partner dynamics that develop over time and resist traditional measurement.

The concept of connective tissue originated through multilateral knowledge exchange and learning exercises between RTI International and the Access Accelerated strategic partners. This analysis marks the first attempt to formalize the concept in peer-reviewed research and illustrate its implications for the field of implementation science. Further research is warranted to refine the concept of connective tissue and its measurement within multisectoral partnerships - not only as an emerging idea in academia and professional practice, but also because of its potential to inform the next generation of global development goals and multisectoral partnerships in global health.

## Methods

This study explores connective tissue using Access Accelerated as a case study through a mixed-methods survey of strategic partner project stakeholders in four countries. The study aims to:

1. Introduce the concept of connective tissue as an explanatory factor in the success and sustainability of multisectoral collaboration,
2. Identify barriers and facilitators to the development of connective tissue, and
3. Assess stakeholder perspectives on the conceptual validity of connective tissue and its potential influence on project outcomes

Project stakeholders are prompted to consider three dimensions of connective tissue including (1) knowledge creation, (2) trust, and (3) social capital, as well as facilitators and barriers of their development within the partner network. For the purposes of this study and its component survey, these concepts are defined in Table 1 as follows:

**Table 1:** Connective tissue and its three dimensions

**Connective Tissue** refers to the social capital, knowledge creation, and trust that arise when multiple partner efforts are directed towards unified goals **Knowledge creation** in this context is the collection and analysis of project information, as well as the transfer and sharing of key insights **Trust** in this context is confidence in the sincerity, reliability, competency and credibility of a partner **Social capital** in this context refers to a set of shared values or resources that allows individuals to work together in a group to effectively achieve a common purpose. Social capital can help an organization to obtain resources, information, and other forms of assistance from their network of partners	

We distributed the survey to 29 stakeholders involved in C/Can, NCDA, PATH, World Bank, and WHF projects funded by Access Accelerated, focusing on implementors of projects in Colombia, Ghana, Kenya, and Vietnam – all countries where at least three of the five strategic partners have implemented projects since 2017, and have thus had ample opportunities to interact with other organizational members. The presence of Access Accelerated strategic partners in these four countries from 2017 to 2022 is shown in Table 2.

**Table 2:** Access accelerated strategic partner presence in selected countries from 2017 to 2022

Strategic Partner	Colombia	Ghana	Kenya	Vietnam
City Cancer Challenge	X	X		
NCD Alliance		X	X	X
PATH		X	X	X
The World Bank	X	X	X	X
World Heart Federation	X		X	

### Distribution and respondents

The survey was distributed by five focal persons representing each of the Access Accelerated strategic partners to staff from their organizations and local stakeholders involved in project implementation in the selected countries. Selection of survey recipients was not comprehensive, and was performed at the discretion of strategic partner focal persons based on the recipients’ location in one of the four focus countries and their role in project implementation entailing interaction with other members of Access Accelerated partner network. This decision was taken to maximize the survey’s response rate, and while distribution of the survey by members of the subjects’ own organization and partner organizations introduces a risk of bias, this bias was mitigated by a protocol whereby disaggregated, individual responses were only accessible to authors who were affiliated with evaluation partner RTI International and unknown to the respondents. These same RTI-affiliated authors also confirmed respondent eligibility under the above criteria.

Survey recipients were informed of how their responses would be analyzed in a brief pre-survey letter. The study followed a consent process which ensured that responses were voluntary, confidential, and would not pose reputational risk to the respondents within their organizations. It was conducted in compliance with EU General Data Protection Legislation. The study was designated as exempt by RTI International’s Institutional Review Board on the grounds that participants’ disclosure did not reasonably place them at risk of criminal or civil liability or damage to their reputation, employability, or financial standing.

**Fig. 1:**
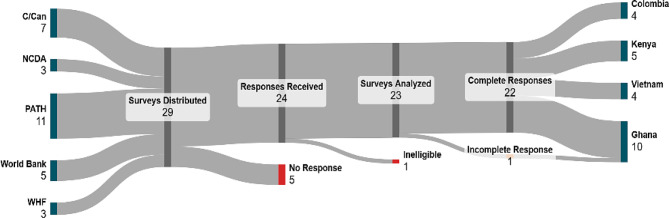
Survey distribution and respondents included in analysis

The survey was distributed online via Microsoft Forms between August 2023 and January 2024, with a Spanish translation available for respondents in Colombia. The survey received initial responses from 24 out of 29 recipients, for a response rate of 83%. One respondent’s input was recorded only for the first section as they did not complete the survey. Another was removed from the sample because they were deemed not to have been a project implementor or stakeholder in one of the four study countries. This distinction is important because, while connective tissue can theoretically form between international and global partners, the survey was designed to explore its effects on collaboration at national level. As such, the total number of surveys included in this analysis is 23. A visualization of survey distribution is included below in Fig. 1.

Despite potential bias that may have been introduced through the participant sampling methods, the study methodology and case study approach are appropriate for an exploratory study with the primary aims of developing a new theory, investigating barriers and facilitators, and recording implementor’s subjective opinions, rather than empirically assessing project success or team performance.

### Survey structure and analysis

The survey consisted of three sections: stakeholders’ views on possible barriers an facilitators, their assessments of connective tissue within the partner network, and their perceptions of potential benefits. Questions were developed by a panel of experts involved in the monitoring and evaluation of the Access Accelerated initiative to ensure applicability and relevance of the survey. Given that “connective tissue” is a new concept, we did not rely on validated qualitative assessments to provide survey questions.

In Section “Background”, hypothesized barriers and facilitators are listed, and participants are asked to describe through multiple choice how present and impactful these factors were in their experience. Participants are to indicate whether they attest to any of those barriers, and are provided a free response to elaborate on their responses and suggest additional facilitators and barriers. Section “Results” includes Likert Scale questions for the three dimensions of knowledge creation, trust, and social capital, prompting participants to provide their level of agreement with statements indicating that these qualities had been cultivated and had positive impacts for their projects. Section “Conclusions” inquires whether respondents attest to any additional benefits of connective tissue. The complete survey is available in Appendix 1.

Data were collected and analyzed by authors affiliated with RTI International who lacked any personal or professional association with the sample of respondents. Complete sections of incomplete surveys (*n* = 1) were included, while incomplete sections were omitted. Quantitative results are reported as raw counts or percentages. Qualitative results are analyzed thematically, with the most representative quotes prioritized for inclusion either to support quantitative findings or illustrate the practical implications of core concepts in relation to project implementation. Write-in responses are reported wherever possible.

Although responses come from four specific focus countries, different partner overlap and project portfolios in each location prohibit head-to-head comparisons between country results. Responses are analyzed and reported in aggregate, as this study’s focus is the experience of a national-level stakeholder within a global multisectoral partnership. One resulting limitation of this study is a lack of consideration for how external factors, such as the cooperative norms within each country’s institutions, may have influenced results. Global initiatives with more directly comparable activities across countries present an opportunity for future research to investigate how connective tissue manifests differently in various geographies.

## Results

Survey responses from 23 project staff and stakeholders were included in the sample for this analysis. Geographically, 4 responses were recorded from Colombia, 10 from Ghana, 5 from Kenya, and 4 from Vietnam. By strategic partner, 3 surveys came respondents nominated by C/Can, 3 from NCDA, 9 from PATH, 5 from the World Bank, and 3 from WHF. C/Can and the World Bank distributed surveys exclusively to their employees in the selected countries, whereas NCDA, PATH, and WHF distributed to local affiliates and implementing partners for their projects in each country. Aside from staff from the five strategic partners, stakeholders and affiliates represented among survey respondents included government health authorities, civil society partners, and non-governmental organizations.

### The three dimensions of connective tissue

#### Knowledge creation

**Fig. 2:**
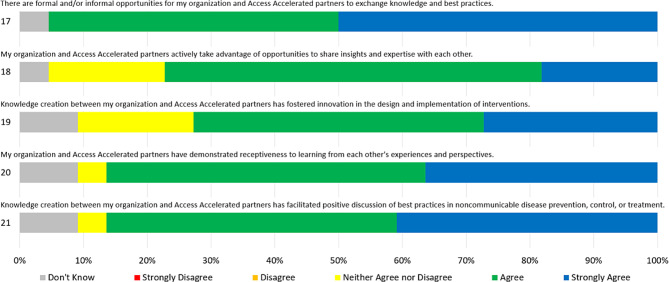
Knowledge creation likert scores

In their answers to Likert Scale prompts on knowledge creation, shown below in Fig. 2, respondents largely indicated that they had witnessed opportunities to exchange knowledge (95% answered “Agree” or “Strongly Agree”), willingness to learn from one another (86%), and increased discussion of best practices (86%). However, fewer respondents agreed that knowledge creation opportunities had been utilized to their full potential (77%) or that they had resulted in innovation within the implementation of interventions (73%).

In open format answers, survey respondents indicated that knowledge sharing between partners had streamlined effective resource use and allocation, with one relating that “we learnt from the experience of the implementation of interventions and tried to align our interventions so they could be compliment to one another.” Another stated that “inputs [from partners] help improve our planning and organization capacity in policy advocacy,” while a third explained how “lessons drawn [from partner projects] provide means for effective planning and implementation of subsequent activities and projects.” Respondents also indicated that knowledge sharing contributed to the visibility of some projects, with one respondent in Ghana describing how “sharing of navigator findings with programs such as EPI has led to enhanced interest to use the navigator beyond NCDs.”

#### Trust

**Fig. 3:**
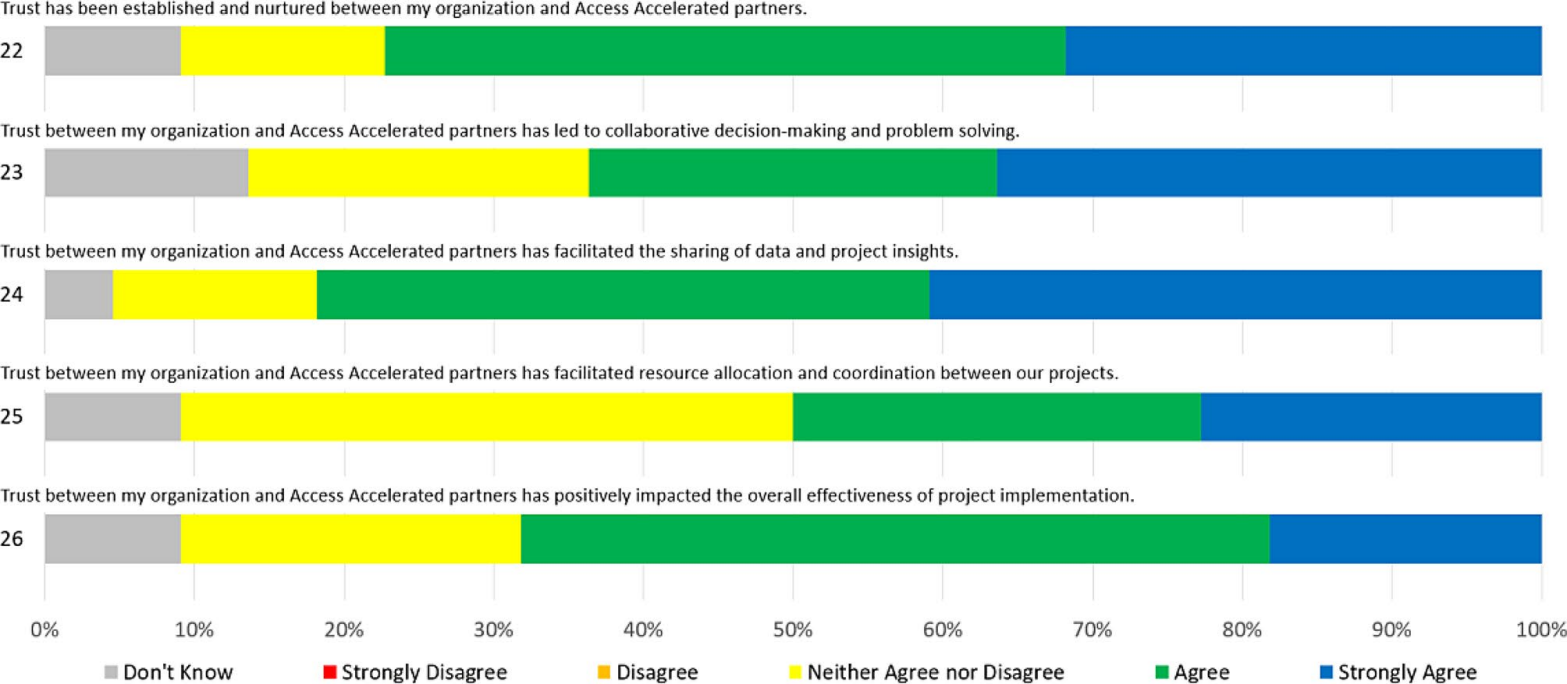
Trust likert scores

Over three-quarters (77%) of respondents expressed moderate or strong agreement that trust had been established and nurtured between partners. Four-fifths (82%) believed that accumulated trust had facilitated the sharing of data and project insights, with half that group expressing strong agreement. On the other hand, just two-thirds (68%) agreed that it had improved overall effectiveness of project implementation, and (64%) that it had contributed to collaborative decision-making and problem-solving. Only half (50%) of respondents indicated any form of agreement that trust facilitated resource allocation. Full Likert Scale responses to prompts on the dimension of trust are shown below in Fig. 3.

Respondent testimony described how trust was facilitated by knowledge sharing and mutual partnerships between stakeholders. One respondent stated that “workshops with participation from relevant partners and units to share knowledge and data increases transparency of government’s work and build trust among partners.” An implementing partner of NCDA explained how that partnership had helped build their network: “We learn the ways NCDA communicates with country partners. We follow that in building trust with Vietnam NCD alliance members.” Strengthening the network of mutual partners is critical to effective implementation, as one respondent emphasized that “sharing local stakeholder connections plays a huge role in fostering trust and ensuring smooth implementation of activities.”

#### Social capital

**Fig. 4:**
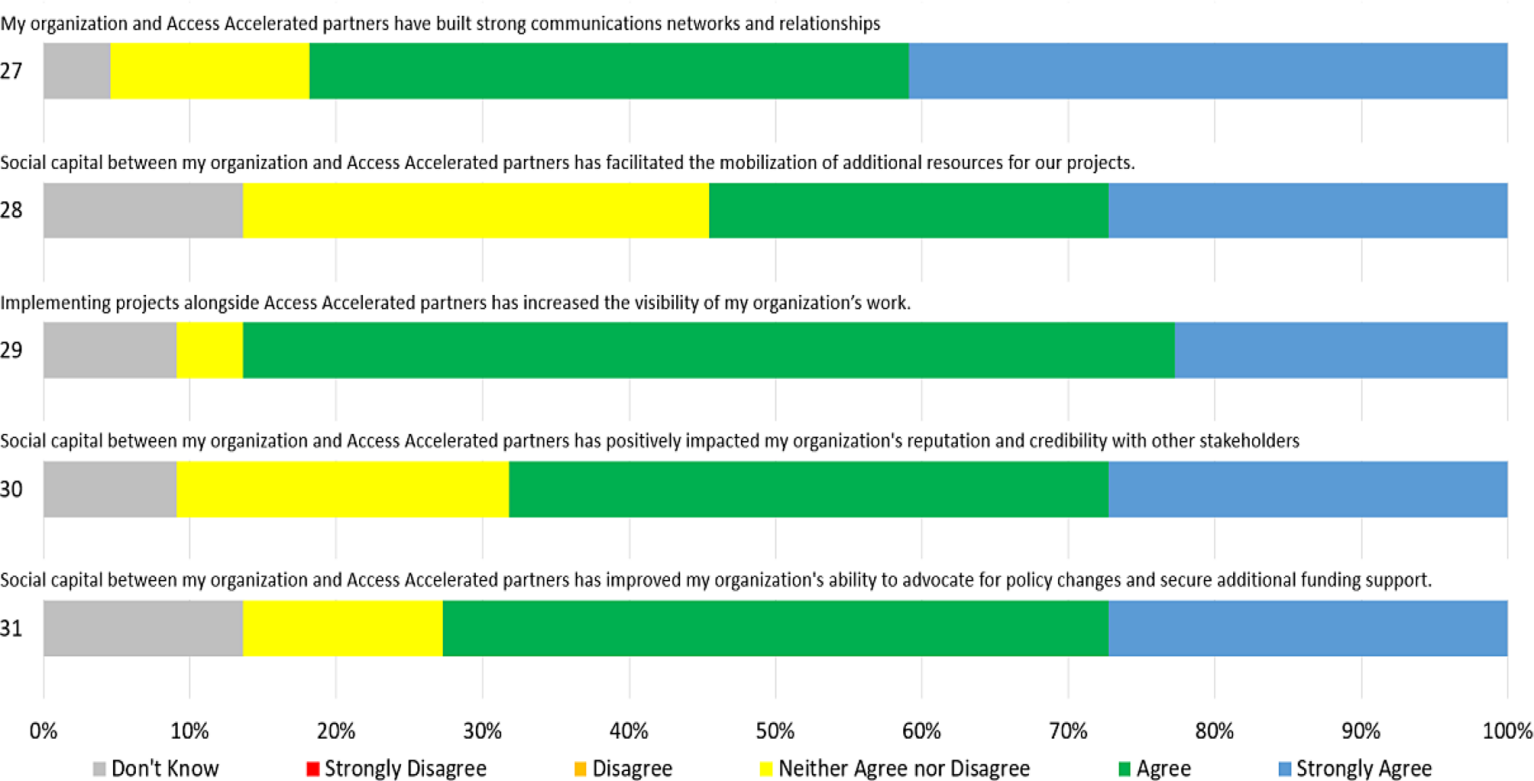
Social capital likert scores

As shown below in Fig. 4, eighty-two percent of respondents indicated agreement that strong communications networks and relationships had been built with Access Accelerated partners, with half that group expressing strong agreement. When considering potential benefits from social capital in the partner network, a strong majority (86%) of respondents reported moderate or strong agreement and that working together had increased the visibility of their projects, compared to majorities nearly three-quarters (73%) agreeing that it had improved their ability to advocate for policy change, two-thirds (68%) that it had had positively influenced their organizational reputation, and just over half (55%) that it had improved their organizational ability to mobilize additional resources.

Open responses clarified how social capital within the Access Accelerated partner network improved opportunities for collaboration and the effectiveness of working relationships. A respondent in Vietnam mentioned how working alongside Access Accelerated helped to “expand social capital across the country partners, including from the government, the party, and the national assembly, at the central and provincial levels.” In Kenya, another explained that “social capital has elevated the profile of our organization, improved our credibility with other stakeholders including the Ministry of Health and fostered a stronger relationship and trust with the [Access Accelerated] partners.” A respondent in Colombia elaborated how “being [Access Accelerated] partners has created the opportunity for local teams to learn from each other’s projects but more interestingly, strengthened personal relationships that allow for more collaboration around the areas we are working towards.”

### Facilitators of connective tissue

Respondents were asked specifically about five factors that could facilitate the three dimensions of connective tissue. These were: 1) similarity in project objectives, 2) the existence of knowledge sharing touchpoints, 3) geographic overlap of project implementation, 4) mutual partnerships, and 5) the passage of time.

Sixteen respondents (70% of total) expressed that similar project objectives had caused some improvement or significant improvement in connective tissue among Access Accelerated partners, while 15 (65%) said the same for knowledge sharing touchpoints. Respondents highlighted that having both knowledge sharing touchpoints and similar project objectives facilitates alignment. For example, one suggested that “prior to the initiation of projects and activities, thorough stakeholder engagements are made to ensure that the agenda is in line with the objectives of our supply chain initiatives. This makes it easy for all the interventions to be apt and aligned with our sector wide objective and arrangements.”

#### Similar project objectives

- *“Having cancer as a common thread allowed strengthening the social capital in at least one of the common geographies where two of these organizations worked. During the interactions, being AA partners created an invisible bond that allowed knowledge sharing and strategy alignment.”*
- *“The Ghana NCD Alliance was instrumental in the NCD Navigator project led by PATH Ghana. While the Navigator identified CSOs and areas of operations, the [Ghana NCD Alliance] identified people with lived experience to support advocacy.”*

#### Knowledge sharing touchpoints

- *“Developing shared materials with learning opportunities and participating in panels organized by either organization has allowed to interact, learn from each other and bring local stakeholders to the spotlight.”*
- *“Larger pool of knowledge shared among partners increase its robustness and integrity, integration of activities also enhances effectiveness and reduce overlapping in activities implemented, overall trust is improved.”*

Only 13 out of 23 (57%) respondents indicated that any improvements in connective tissue could be attributed to mutual partnerships between their organization and other partners, and the same proportion attributed improvements to geographic overlap of partner projects. Respondents also commented on changes in knowledge creation, trust, and social capital over time, indicating that these qualities had strengthened within the working relationships of Access Accelerated strategic partners and stakeholders from 2017 to 2023. The survey elicited the following illustrative quotes from respondents on the ways in which these potential facilitators of connective tissue have impacted their projects:

#### Geographic overlap

- *“Geographic overlap of office and staff enhances the cooperation between partners, increasing both trust and knowledge sharing”*
- *“The fact that the two organizations work together in the same territory strengthens and gives synergy to actions and projects.”*

#### Mutual partnerships

- *“Partnerships can be official or unofficial, and from my perspective with AA partners, mutual partnerships create an unofficial relationship that can be leveraged to either become an official partnership or a common starting point.”*
- *“We meet at events and share our individual and sometimes collective advocacy priorities for government consideration.“*

#### Time

- *“Trust has increased over time.”*
- *“The more time passes, the stronger these bonds become and the more comfortable teams feel to reach out for support, advice or stakeholder management.”*

Respondents suggest that working in the same place at the same time is important and supports collaboration. One noted that an “invisible bond” had resulted from shared objectives and helped develop knowledge sharing, trust, and social capital among partners. Overall, respondents indicate that membership in this network facilitated the mutually beneficial sharing of project insights, advocacy priorities, and local stakeholder connections.

### Identification of barriers to connective tissue

The survey inquired about barriers to cooperation, prompting respondents with a free response option in addition to a checklist. Of the 23 respondents, only 6 (26%) suggested that any of the options presented had hindered cooperation. However, two of the 23 surveys indicated that “geographic distance” had hindered cooperation, as did 3 for “competition for funding,” 3 for “differing goals and priorities,” and 4 for “resource constraints.”

**Fig. 5:**
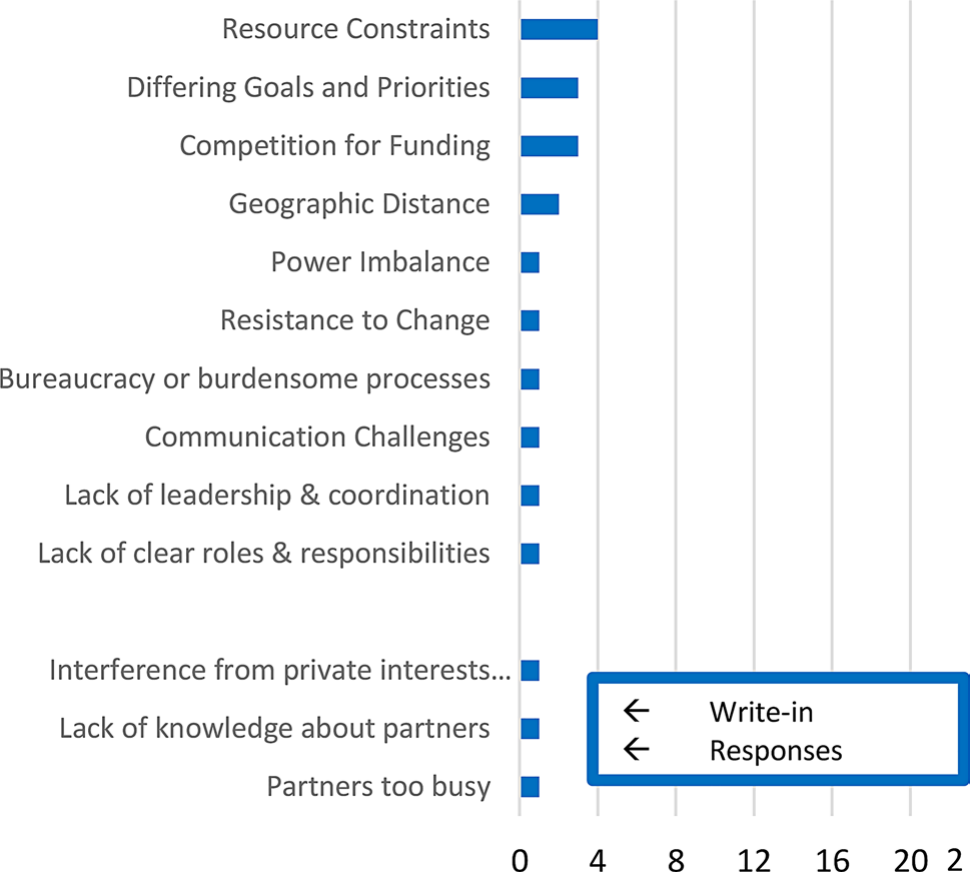
Count of identified barriers to connective tissue

Respondents also mentioned busy schedules among partners, lack of knowledge about partners, and interference from alcohol and tobacco industry groups as barriers. The identification of interference from private sector actors highlights the potential for conflicts of interest within public-private partnerships and underscores the importance of strong governance mechanisms within multisectoral coalitions. Although respondents on average identified less than one barrier to the development of connective tissue, cumulative counts of each response are shown below in Fig. 5.

### Identification of potential benefits from connective tissue

Respondents were prompted to comment on 10 potential benefits from connective tissue as identified by the review panel. The average respondent agreed with 8 of the options presented, with the most frequently cited benefits, “fostered a collective vision for improving access to NCD care” and “created opportunities for stakeholders to identify and leverage each other’s strengths, resources, and expertise,” each being selected by 20 out of 22 (91%) of respondents. Additionally, respondents wrote in that they had seen knowledge creation, trust, and social capital among partners lead to capacity building, the implementation of best practices, elevated organizational profiles, lower government corruption, and meaningful involvement of stakeholders. The collected responses don’t confirm the existence of these benefits or conclusively attribute them to knowledge creation, trust, and social capital, but they reinforce the usefulness of the connective tissue concept and merit further exploration through studies with more rigorous research methods.

## Discussion

In this mixed methods analysis of an Access Accelerated stakeholder survey, respondents described positive impacts from multisectoral collaboration that go beyond the stated project goals. Implementation science methods can measure connective tissue, the less tangible qualities of collective efforts that bind stakeholders and activities together, build coherence across multiple objectives and ways of working, and lead to sustainable actions over time. While the present study proposes knowledge creation, trust, and social capital as critical dimensions of connective tissue, future research may suggest additional organizational qualities that enhance the effectiveness and sustainability of partnerships.

This four-country survey suggests that connective tissue can be developed at local and national levels, which holds significant implications for measurement approaches within global multisectoral partnerships. This study explores the perspectives of project implementors and stakeholders, but subjective survey responses are insufficient to conclusively assess the material outcomes of connective tissue. It is possible that the outcomes of connective tissue can be better studied from a health systems perspective, and future research with more rigorous methods is encouraged to further refine the theory. Nonetheless, the mixed methods and case study approach applied here provide a valuable foundation for the measurement of connective tissue by identifying factors that influence knowledge sharing, trust, and social capital within a partner network.

### Facilitators and barriers

This study indicates that several factors can act as facilitators to the creation of connective tissue. When partners target shared objectives as opposed to adjacent ones, they can participate in joint planning and materials development, which strengthens organizational ties. Geographic proximity opens the doors for deeper engagement and knowledge sharing opportunities. Finally, when two aligned organizations also have a mutual partnership with a third organization such as the government health authority, this further increases trust between partners and leads to more robust knowledge creation. The larger pool of local knowledge and resources to draw from helps leverage stakeholder strengths and enhance effectiveness of project implementation. Results from this study echo previous findings that shared goals, trust building, knowledge sharing, and other aspects of partnership culture represent valid indicators for intersectoral collaboration in collective efforts for health promotion [16].

The primary barriers cited by respondents were financial constraints and resource allocation. Responses indicated that social capital and trust between partners have limited impact on the mobilization, allocation, and coordination of resources. When asked how resource scarcity had affected project implementation, one respondent expressed that “limited funding only allows for certain activities to be implemented, [when] there are many more could be done with more resources allocated.” Previous research by McCullough et al. (2020) cites lack of trust and resource competition as barriers to interorganizational collaboration [5]. Stronger collaboration in theory can improve resource allocation and reduce scarcity, but survey responses in the present study suggest that connective tissue has not preempted concerns about resource scarcity in all cases within the Access Accelerated partner network.

### Implications for implementation science and measurement of multisectoral collaboration

The concept of connective tissue builds off and complements existing frameworks to measure multisectoral collaboration but incorporates implementation science recommendations to apply adaptable and mixed-methods approaches. For instance, while Kania and Kramer’s five conditions of collective impact are baked into the governing structure of a partnership, the dimensions of connective tissue develop over time and evolve in concert with shifting project, policy, and partnership dynamics. Whereas the collective impact framework identifies structural and foundational elements of social initiatives which enable effective collaboration, the concept of connective tissue seeks to identify and measure more fluid elements which exert similar influence and help partnerships to achieve shared goals.

The application of connective tissue in practice produces three key implications for future measurement of multisectoral partnerships and collective action campaigns. First, the concept of connective tissue is helpful in demonstrating the value additions created by different stakeholders working together. Breakthroughs in partnership can occur at any stage of project implementation, and greater understanding of connective tissue’s potential benefits can encourage stakeholders to prioritize collaboration over competition. Secondly, from a programmatic perspective, there is value in defining progress that may not be captured within logical frameworks, but that nonetheless affects the success and achievements of outcome indicators. Lastly, connective tissue enables us to understand how quantifiable results may be linked to one another, which is not always possible through traditional measurement systems. This is important insofar as it allows a more nuanced understanding of how projects may be integrated, including through shared funding, coordinated implementation, or mutual partnerships.

The SDGs brough forth a flourishing era of multilateral assistance, along with innovative partnerships such as Gavi and the Global Fund. However, development assistance for health hit a high water mark in 2021 amidst the COVID-19 pandemic, and have now fallen to their lowest levels since 2009 [17]. As historical donors withdraw their support, many low- and middle-income country are under pressure to mobilize greater resources for health. The December 2023 Lusaka Agenda already calls for shifts in strategic coordination, sustainable domestic health financing, and stronger joint approaches to improve health outcomes [18]. As the SDG era of development assistance winds down and health systems face mounting resource constraints, it is more important than ever before that Ministries of Health, development banks, private sector and nonprofit implementing partners find effective models of multisectoral collaboration. These qualitative findings on connective tissue can contribute to design, implementation, and monitoring of the next generation of multisectoral partnerships for health, particularly those that support sustainable systems strengthening.

### Limitations

There are limitations to our mixed methods, case study approach and presentation of connective tissue. Notably, the data presented are insufficient to empirically measure connective tissue or conclusively determine that it has yielded benefits in organizational or project outcomes. Further, while surveying perspectives of project stakeholders produces strong qualitative insights into factors that influence the quality of multisectoral collaboration, this exploratory analysis does not comprehensively address all potential barriers, facilitators, and dimensions of connective tissue. Future research in the field of implementation science can expand the concept of connective tissue beyond the dimensions of knowledge sharing, trust and social capital, identify additional factors influencing its development, and more concretely establish beneficial effects within partner networks and health systems.

Another important limitation of this study is the role of authors affiliated with Access Accelerated strategic partners in distribution of the survey. To maximize response rate among respondents, the Microsoft Forms survey link was distributed by authors affiliated with C/Can, NCDA, PATH, WHF and the World Bank. Distribution of the survey through known colleagues or associates of respondents may have invited bias in respondents’ answers. However, a data analysis protocol was established and communicated to respondents whereby individual and disaggregated survey responses were made available only to authors affiliated with RTI International, with whom respondents had no association. This study also sought to control potential bias by allowing for neutral and non-response options in all cases.

Respondents were not selected randomly from a predefined pool of stakeholders for projects supported by Access Accelerated. Rather, they were nominated because of their geographic location and level of interaction with representatives of other partner organizations. The four countries in which surveys were conducted were chosen on the basis of geographic and temporal overlap of partner activities and are not representative of the entire portfolio of Access Accelerated across more than thirty countries. Finally, our analysis of connective tissue is essentially retrospective. While Access Accelerated and its strategic partners have implemented projects and cultivated relationships in low- and middle-income countries since 2017, the concept of connective tissue was not ideated until 2021, and the survey on which this study is based occurred in 2023.

Our findings are presented here as key insights and lessons learned through the implementation experience of Access Accelerated, and merit more rigorous assessment by subsequent evaluations of multisectoral collaborations in global health. Our aim is to put forth the concept, illustrate its potential value in measurement of multisectoral partnership, and encourage its further exploration through future implementation science research, including by piloting an example of how to pursue such investigation. Future research can validate the concept of connective tissue by measuring the development of knowledge creation, trust, and social capital between partners over longer periods of time; further identifying barriers, facilitators, and effects of connective tissue; demonstrating interventions that effectively build connective tissue; and linking connective tissue more concretely to improved project outcomes.

## Conclusions

Capturing specific project achievements through traditional measurement frameworks limits our ability to see how accomplishments build on each other and reinforce collaborative and effective working relationships among partners. Whole-of-society approaches to public health and other social causes require methods which assess the strength of partnership between different actors working cohesively to achieve shared goals. Stakeholder surveys, an important tool borrowed from the field of implementation science, provide one option to inform future measurement approaches by capturing real-time evidence regarding knowledge creation, trust, and social capital among partners. For instance, findings from this study support strong investment in structures for communications, knowledge sharing, and monitoring and evaluation within multisectoral coalitions.

Identifying ways of capturing the impacts from collective action – both tangible and intangible – is an understudied area. Attention to the cultivation of connective tissue between partners helps reimagine the less tangible but potentially impactful effects of multiple stakeholders aligning their efforts towards a shared vision. As the shifting global health financing landscape forces reconsideration of national and international priorities, effective multisectoral collaboration is more important now than ever before.

## Electronic supplementary material

Below is the link to the electronic supplementary material.

Supplementary Material 1

## Data Availability

The data that support the findings of this study are available from the corresponding author upon reasonable request and with permission of Access Accelerated.

## Abbreviations

SDGs: Sustainable Development Goals
NCDs: Noncommunicable diseases
C/Can: City Cancer Challenge
NCDA: Noncommunicable Disease Alliance
WHF: World Heart Federation
CSO: Civil Society Organization

## Footnotes

1 C/Can works with cities globally to increase access to equitable and quality cancer care through a collaborative and locally-led process. NCDA advocates globally for action on NCDs, promotes the meaningful involvement of people living with NCDs in policy processes and strengthens advocacy capacity of national and regional alliances through capacity development programs. PATH is a global non-profit dedicated to achieving health equity through innovation and partnerships targeting pressing health challenges, and building the evidence to improve access to prevention, treatment, and care for people living with NCDs through integrated, people-centered primary health care models. The World Bank works through national governments to develop health solutions and create enabling environments for their implementation. WHF strengthens delivery of high-quality cardiovascular disease treatment, care and prevention, and advocates for cardiovascular health at the national and global levels.
