# Supplementary material for: Assessing ‘connective tissue’ in public-private partnerships: a stakeholder survey on multisectoral collaboration in global health
Source: Global Health. 2025 Nov 11;21:67. doi: 10.1186/s12992-025-01156-x (PMC12607086; doi:10.1186/s12992-025-01156-x)
Supplement: Supplementary file 1 — Supplementary Material 1 [file 12992_2025_1156_MOESM1_ESM.docx]

**Appendix A: Connective Tissue Survey**

**Survey Description:**

*This survey contains 50 questions and takes approximately 30 minutes to complete. After a few introductory questions about your role within Access Accelerated projects, the survey consists of 3 sections examining different aspects of connective tissue. Section 1 assess factors that can hinder or contribute to the formation of connective tissue. Section 2 assesses levels of social capital, knowledge creation, and trust which exist between your organization and Access Accelerated Partners. Section 3 inquires whether you can attest to any tangible benefits that have arisen from the presence of connective tissue.*

**Definitions:**

**Connective Tissue** refers to the social capital, knowledge creation, and trust that arise when multiple partner efforts are directed towards unified goals.

**Social capital** in this context refers to a set of shared values or resources that allows individuals to work together in a group to effectively achieve a common purpose. Social capital can help an organization to obtain resources, information, and other forms of assistance from their network of partners.

**Knowledge creation** in this context is the collection and analysis of project information, as well as the transfer and sharing of key insights.

**Trust** in this context is confidence in the sincerity, reliability, competency and credibility of a partner.

**Access Accelerated Partners** include City Cancer Challenge (C/Can), Noncommunicable Disease Alliance (NCDA), PATH, the World Bank, and the World Heart Federation.

**Respondent Information:**

1. Respondent Name: ________________________________________________________________________________________________
2. Respondent Organization: ___________________________________________________________________________________________
3. Respondent Job Title: ______________________________________________________________________________________________
4. Respondent Email: _________________________________________________________________________________________________
5. Please briefly describe your role in Access Accelerated projects implementation (alongside City Cancer Challenge, NCD Alliance, PATH, the World Bank, and/or World Heart Federation).____________________________________________________________________________

**Section 1 - Facilitators of connective tissue:**

*The following questions pertain to your knowledge and perspectives of the project activities of Access Accelerated partners. The questions will assess factors that can hinder or contribute to the formation of connective tissue, including geographic overlap, mutual partnerships, knowledge sharing, and time.*

Mutual objectives and activities:

6. From your perspective, are Access Accelerated Partner projects targeting objectives that are similar to those that are targeted by your organization?

Not similar Somewhat similar Very similar [I don’t know/no answer]

7. From your perspective, are Access Accelerated Partner projects conducting activities that are similar to the type of activities conducted by your organization?

Not similar Somewhat similar Very similar [I don’t know/no answer]

8. Have similarities in project objectives and activities affected levels of trust, knowledge creation, and/or social capital between your organization and Access Accelerated partners?

Harmful effect No change Some improvements Significant improvements [I don’t know/no answer]

9. Please describe the ways in which similarities in project activities have affected the levels of trust, knowledge creation, and/or social capital between your organization and Access Accelerated partners.

[Open response]: ______________________________________________________________________________________________________

_____________________________________________________________________________________________________________________

Geographic overlap:

10. To your knowledge, which Access Accelerated Partners work in the same geographic areas as your organization? (There is no need to select your own organization, if applicable)

[checklist of 5 Access Accelerated Partners]

11. (for each partner indicated) Since 2017, have you interacted with the above organizations regarding the implementation of projects funded by Access Accelerated? How have these interactions influenced the implementation of either organizations' projects?

[Open response]: ______________________________________________________________________________________________________

_____________________________________________________________________________________________________________________

12. Has geographic overlap with Access Accelerated partner projects affected the levels of trust, knowledge creation, and social capital between your organization and these partners?

Harmful Effect No change Some improvements Significant improvements [I don’t know/no answer]

13. Please describe how geographic overlap with Access Accelerated partner projects has affected the levels of trust, knowledge sharing, and social capital between your organization and these partners.

[Open response]: ______________________________________________________________________________________________________

_____________________________________________________________________________________________________________________

Mutual Partnerships:

14. To your knowledge, are Access Accelerated Partners in your country working directly with any organizations (government, private sector, nonprofit, community groups, etc) that you also work with? Which organizations?

[Open response]: ______________________________________________________________________________________________________

_____________________________________________________________________________________________________________________

15. Has the existence of mutual partners between your organization and Access Accelerated Partners affected the levels of trust, knowledge sharing, and/or social capital between project stakeholders?

Harmful effect No change Some improvements Significant improvements [I don’t know/no answer]

16. Please describe how mutual partnerships between your organization and Access Accelerated partners have affected the levels of trust, knowledge sharing, and/or social capital between project stakeholders.

[Open response]: ______________________________________________________________________________________________________

_____________________________________________________________________________________________________________________

Knowledge Sharing Touchpoints:

17. To your knowledge, does your organization utilize any shared measurement systems with any Access Accelerated Partners to capture project indicators? (No need to select your own organization)

[checklist of 5 Access Accelerated Partners]

18. To your knowledge, has your organization engaged in any formal knowledge sharing touchpoints (such as project debriefs, capacity building workshops, data and intelligence coordination sessions) with representatives of Access Accelerated Partners since 2017?

No knowledge sharing touchpoints One or two knowledge sharing touchpoints Three or more knowledge sharing touchpoints

19. Have knowledge sharing touchpoints affected the levels of trust and/or social capital between your organization and Access Accelerated partners?

Harmful effect No change Some improvements Significant improvements [I don’t know/no answer]

20. Please describe the ways in which knowledge sharing touchpoints have affected the levels trust and/or social capital between your organization and Access Accelerated partners.

[Open response]: ______________________________________________________________________________________________________

_____________________________________________________________________________________________________________________

Time:

21. How have your individual and organizational relationships with Access Accelerated Partners changed since 2017? Has this change over time influenced any of your earlier responses?

[Open response]: ______________________________________________________________________________________________________

_____________________________________________________________________________________________________________________

Barriers:

22. To your knowledge, have any of these common barriers hindered cooperation between your organization and Access Accelerated partners? (mark all that apply)

Communication challenges Lack of trust Differing goals and priorities Power imbalance Bureaucracy or burdensome processes Resource constraints Competition for funding Cultural differences Lack of leadership and coordination
Lack of clear roles and responsibilities Resistance to change Geographic distance None of the above have hindered cooperation

23. Please describe how the above barriers have hindered cooperation between your organization and Access Accelerated Partners.

[Open response]: ______________________________________________________________________________________________________

_____________________________________________________________________________________________________________________

Other barriers and facilitators:

24. Are there any other factors which have hindered or contributed to the development of trust, knowledge creation, and/or social capital between your organization and Access Accelerated partners since 2017? What other factors have been influential?

[Open response]: ______________________________________________________________________________________________________

_____________________________________________________________________________________________________________________

**Section 2 – Aspects of Connective Tissue:**

*Questions in this section will ask for your personal assessments of the levels of social capital, trust, and knowledge creation which exist between your organization and Access Accelerated Partners.*

*Each question can be answered using a Likert Scale with the following options:*

*[I don’t know] Strongly Disagree Disagree Neither agree nor disagree Agree Strongly Agree*

Social Capital:

25. My organization and Access Accelerated partners have built strong communications networks and relationships.

26. Social capital between my organization and Access Accelerated partners has facilitated the mobilization of additional resources for our projects.

27. Implementing projects alongside Access Accelerated partners has increased the visibility of my organization’s work.

28. Social capital between my organization and Access Accelerated partners has positively impacted my organization's reputation and credibility with other stakeholders (funders, government, potential partners, community groups).

29. Social capital between my organization and Access Accelerated partners has improved my organization's ability to advocate for policy changes and secure additional funding support.

Trust:

30. Trust has been established and nurtured between my organization and Access Accelerated partners.

31. Trust between my organization and Access Accelerated partners has led to collaborative decision-making and problem solving.

32. Trust between my organization and Access Accelerated partners has facilitated the sharing of data and project insights.

33. Trust between my organization and Access Accelerated partners has facilitated resource allocation and coordination between our projects.

34. Trust between my organization and Access Accelerated partners has positively impacted the overall effectiveness of project implementation.

Knowledge Creation:

35. There are formal and/or informal opportunities for my organization and Access Accelerated partners to exchange knowledge and best practices.

36. My organization and Access Accelerated partners actively take advantage of opportunities to share insights and expertise with each other.

37. Knowledge sharing between my organization and Access Accelerated partners has fostered innovation in the design and implementation of interventions.

38. My organization and Access Accelerated partners have demonstrated receptiveness to learning from each other's experiences and perspectives.

39. Knowledge sharing between my organization and Access Accelerated partners has facilitated positive discussion of best practices in noncommunicable disease prevention, control, or treatment.

**Section 3 – Benefits of Connective Tissue:**

*Questions in this section will explore whether the aspects of connective tissue explored above have resulted in tangible benefits to project processes, outcomes, or implementing organizations.*

*Responses in this section follow a multiple-choice format with options including “yes,” “no,” “don’t know,” and “no response.” Please choose the option which corresponds with your understanding of how projects have benefitted from the presence of connective tissue between stakeholders.*

Connective Tissue & Project Outcomes:

40. From your perspective, have social capital, trust, and knowledge creation between my organization and Access Accelerated partners fostered a collective vision for improving access to NCD care?

41. From your perspective, have social capital, trust, and knowledge creation between your organization and Access Accelerated partners facilitated the creation of platforms or mechanisms that foster ongoing dialogue and coordination among stakeholders?

42. From your perspective, have social capital, trust, and knowledge creation between your organization and Access Accelerated partners facilitated commitment to long-term collaboration beyond current project durations?

43. From your perspective, have social capital, trust, and knowledge creation between your organization and Access Accelerated partners created opportunities for stakeholders to identify and leverage each other's strengths, resources, and expertise?

44. From your perspective, have social capital, trust, and knowledge creation between your organization and Access Accelerated partners led to local government and/or community ownership of NCD projects?

45. From your perspective, have social capital, trust, and knowledge creation between your organization and Access Accelerated partners led to the identification and implementation of cost-saving strategies?

46. From your perspective, have social capital, trust, and knowledge creation between your organization and Access Accelerated partners led to project expansion and/or scale-up?

47. From your perspective, have social capital, trust, and knowledge creation between your organization and Access Accelerated partners improved the sustainability and long-term viability of projects?

48. From your perspective, have social capital, trust, and knowledge creation between your organization and Access Accelerated partners led to improved project outcomes?

49. From your perspective, have social capital, trust, and knowledge creation between your organization and Access Accelerated partners produced notable results that would not have been achieved without such partnership?

50. From your perspective, have social capital, trust, and knowledge creation between my organization and Access Accelerated partners led to any other tangible benefits that were not mentioned previously? If so, what?

[Open response]: ______________________________________________________________________________________________________

_____________________________________________________________________________________________________________________
